# A Novel Method of Identifying Paddy Seed Varieties

**DOI:** 10.3390/s17040809

**Published:** 2017-04-09

**Authors:** Kuo-Yi Huang, Mao-Chien Chien

**Affiliations:** Department of Bio-Industrial Mechatronics Engineering, National Chung Hsing University, Tai-Chung 402, Taiwan; posoda2115123@gmail.com

**Keywords:** paddy seeds, image processing, identification

## Abstract

This paper presents a novel method for identifying three varieties (Taikong 9, Tainan 11, and Taikong 14) of foundation paddy seeds. Taikong 9, Tainan 11, and Taikong 14 paddy seeds are indistinguishable by inspectors during seed purity inspections. The proposed method uses image segmentation and a key point identification algorithm that can segment paddy seed images and extract seed features. A back propagation neural network was used to establish a classifier based on seven features that could classify the three paddy seed varieties. The classification accuracies of the resultant classifier for varieties Taikong 9, Tainan 11, and Taikong 14 were 92.68%, 97.35% and 96.57%, respectively. The experimental results indicated that the three paddy seeds can be differentiated efficiently using the developed system.

## 1. Introduction

Paddy is one of the main crops in Taiwan and can be planted twice each year. Purity analysis is crucial for nurseries and farmers, and purity is determined by paddy variety inspection. Purity is defined by professional inspectors according to the paddy’s appearance, shape, and color. However, there are approximately 500 cases (every case including approximately 4000 paddy seeds) of incorrect purity analyses every year in Taiwan. The jobs of inspection burden the inspectors with loading. Because healthy seedlings from seedling propagation stations (nurseries) are used to cultivate fields of paddy, seed quality is a critical factor when growing seedlings.

Image processing is widely used to inspect grain. MousaviRad et al. [1] used a scanner to capture images of five Iranian rice kernel varieties and then extracted 41 shape features using image processing. The k-nearest neighbors algorithm, a support vector machine, and a backpropagation neural network (BPNN) were used for classification. The classifications of the support vector machine and BPNN were favorable, with accuracies of 97% and 96%, respectively. Mebatsion et al. [2] used a least-squares classifier to identify five varieties of grain through their shape and color features, which were extracted using image processing. The average accuracy of classification was 99.6%. Kuo et al. [3] used image processing and sparse-representation-based classification to distinguish between 30 varieties of rice grains. However, the appearances of these rice grains apparently differ.

Machine learning has been widely used in the establishment of classification mechanisms. Lee et al. [4] used a CCD (charge-coupled device) camera to capture seven varieties of grain kernel and extract 10 shape features and 4 color features using image processing. A BPNN was established in four forms with features identified using principal component analysis and linear discriminant analysis (LDA). The performance of the BPNN with one hidden layer and features in six dimensions (as identified using LDA) was favorable and its classification accuracy was 95%. Sun et al. [5] compared the advantages of a BPNN and a learning vector quantization network (LVQN) for the identification of thermal fuses. The results demonstrated that a BPNN with 20 hidden layer nodes, a learning rate of 0.01, and a tangent sigmoid transfer function exhibited good classification performance with a classification accuracy of 98.0%. However, the LVQN with 160 hidden layer nodes and a learning rate of 0.1 had 91% accuracy. Additionally, Huang [6] presented a BPNN classifier for sorting the quality of areca nuts according to geometric features. Zhang et al. [7] proposed an improved probabilistic neural network for classifying remote-sensing images. Subsequently, Zhang et al. [8] used a fitness-scaled chaotic artificial bee colony (FSCABC) algorithm and feedforward neural network (FNN) to classify fruit. The results showed that the accuracy of the FSCABC–FNN was higher than that of the genetic algorithm–FNN (84.8%), particle swarm optimization–FNN (87.9%), artificial bee colony algorithm (85.4%), and kernel support vector machine (88.2%).

**Figure 1:**
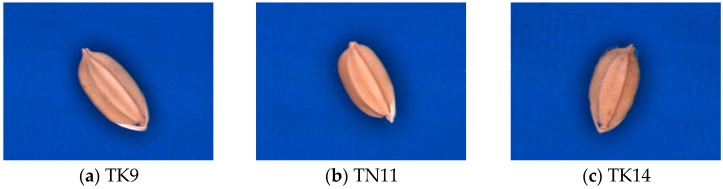
Three paddy seed varieties.

The rice grains examined in previous studies [1,2,3,4] have different appearances and are more distinguishable than the Taikong 9 (TK9), Tainan 11 (TN11), and Taikong 14 (TK14) seeds, which were the focus of the present study. TK9, TN11, and TK14 are so similar on appearance that they are difficult to identify (Figure 1). Therefore, the purpose of this study was to establish an algorithm for recognizing these three paddy seeds. Specifically, the geometric features of the seeds were to be extracted and then used to differentiate between the three paddy seed varieties (TK9, TN11, and TK14).

## 2. Materials and Methods

### 2.1. Image Capture System and Experimental Samples

The image capture system developed in this study comprised a USB CCD color camera (DFK-21BU04, ImagingSource Inc., Taipei, Taiwan), a low-distortion lens (ML-MC25HR, MORITEX Inc., Saitama, Japan), a shadowless lamp (MSRL-CW33, MORITEX Inc.), and a computer (Intel Core i5-4460 CPU, 3.26 GB of RAM, Santa Clara, CA, USA). It captured RGB color images measuring 640 × 480 pixels in the bitmap format. The CCD camera was employed for image acquisition with 28,200 lx and the work distance was 11.0 cm. Image processing software was developed using Visual Basic 6.0 and the Matrox Imaging Library (MIL) 8.0. Paddy seeds (which were foundation seeds in 2014)—varieties TK9, TN11, and TK14 (Figure 1)—were provided by the Taiwan Seed Improvement and Propagation Station.

### 2.2. Image Segmentation

**Figure 2:**
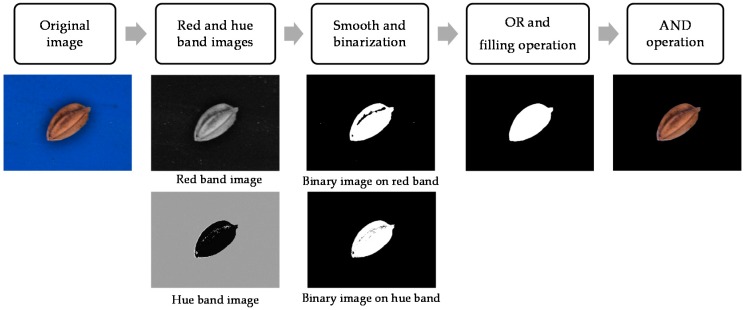
Segmentation procedure.

Segmenting of the paddy seed images is an essential procedure once the features of the paddy seeds have been extracted. The segmentation steps and results (Figure 2) are thus described:

Step 1 Red and hue band images are obtained from the original image.
Step 2 Red and hue band images are treated using a smoothing operator and converted into binary images with an optimum threshold value using Otsu’s method [9].
Step 3 Complete paddy seed binary images are obtained using the OR logic operator and filling operator on the hue and red binary images, respectively.
Step 4 The entire segmented image is obtained using the AND operator on the binary and original images.

In this study, paddy seeds were auto-segmented according to these steps:

### 2.3. Feature Extraction

**Figure 3:**
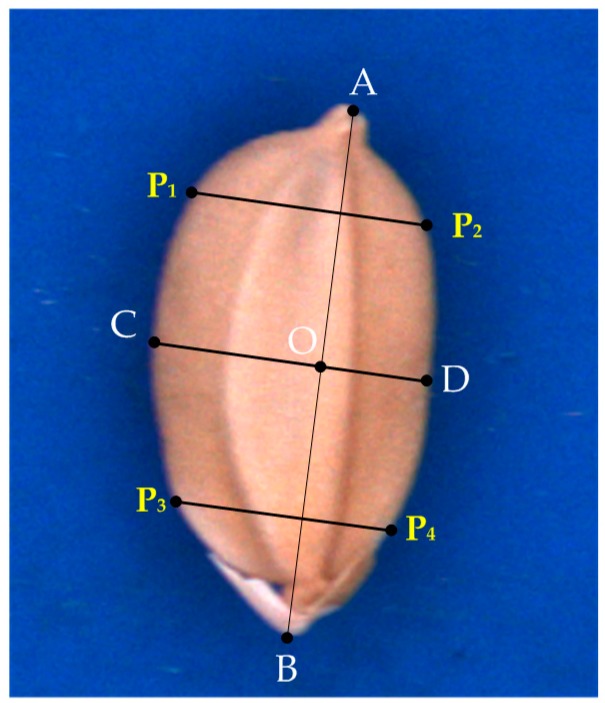
Key lines in the seed contour.

**Figure 4:**
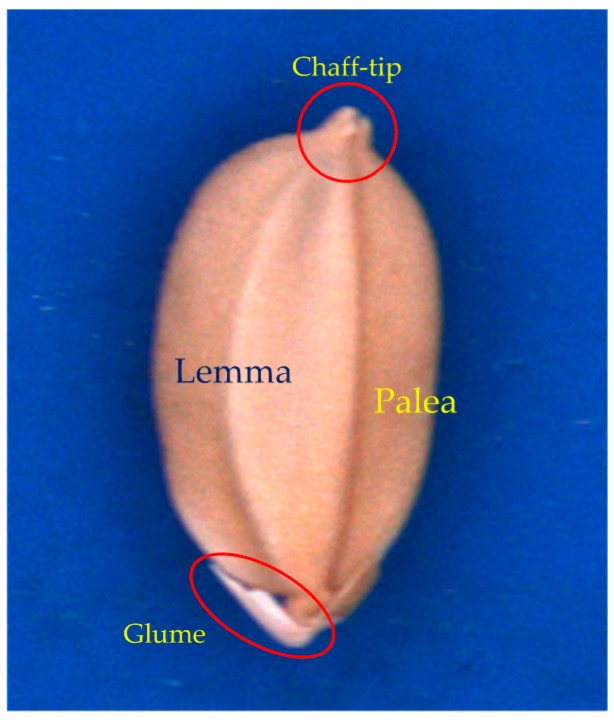
Lemma, palea, glume, and chaff tip of the seed.

Key lines (Figure 3) defined by the contour of a seed are related to the geometric features of the seed. $\bar{\mathrm{AB}}$, $\bar{\mathrm{CD}}$, $\bar{\mathrm{CO}}$, $\bar{\mathrm{DO}}$, $\bar{P_{1}P_{2}}$, $\bar{P_{3}P_{4}}$, and O were identified according to a contour-following algorithm [9]. The features of the seeds are defined as follows:

- The lemma, palea, glume, and chaff tip of a seed are illustrated in Figure 4.
- $\bar{\mathrm{AB}}$ is the longest line segment in the seed contour.
- O is the midpoint of $\bar{\mathrm{AB}}$.
- $\bar{\mathrm{CD}}$ is the perpendicular bisector of $\bar{\mathrm{AB}}$, and thus O is the intersection of $\bar{\mathrm{AB}}$ and $\bar{\mathrm{CD}}$.
- $\bar{\mathrm{CO}}$ crosses the lemma.
- $\bar{\mathrm{DO}}$ crosses the palea.
- $\bar{P_{1}P_{2}}$ is the perpendicular line crossing the 1/5 position of $\bar{\mathrm{AB}}$.
- $\bar{P_{3}P_{4}}$ is the perpendicular line crossing the 4/5 position of $\bar{\mathrm{AB}}$.

**Figure 5:**
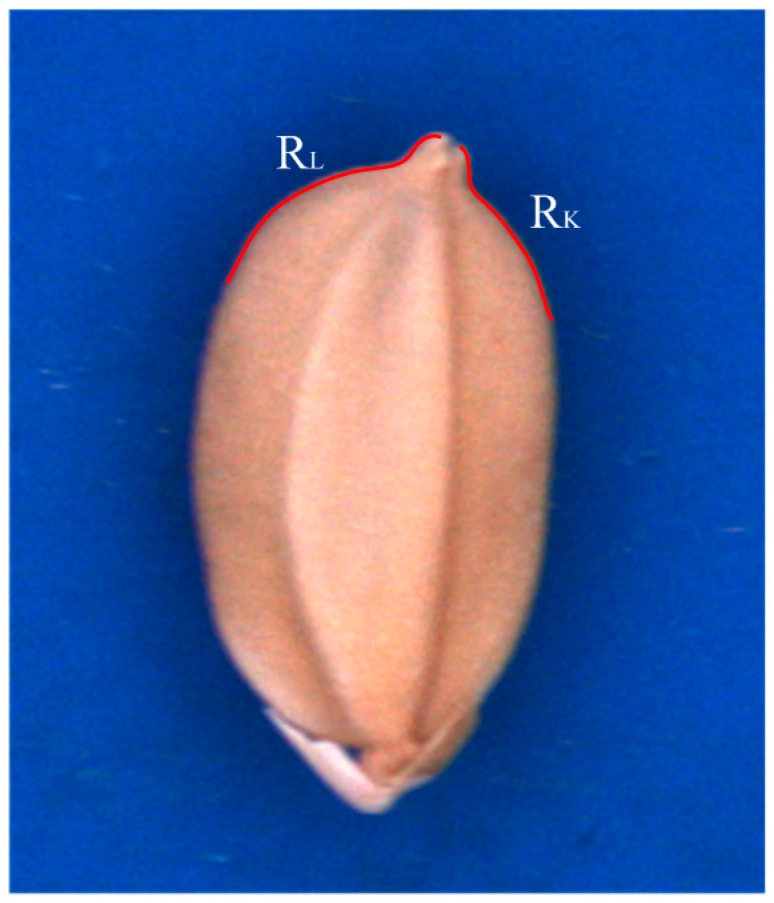
Two concaves at the chaff tip.

**Figure 6:**
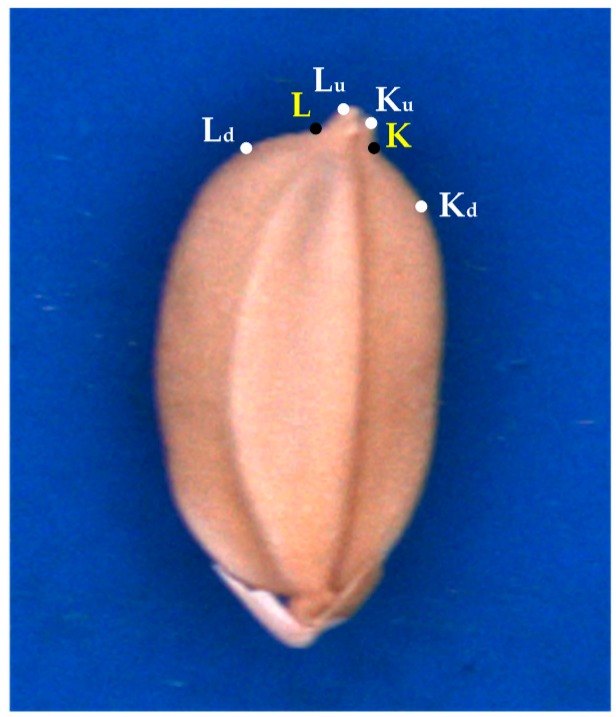
Crucial points on the concaves.

In this study, several special geometric features were extracted to identify seed varieties TK9, TN11, and TK14. Two concaves (R_K_ and R_L_) to the side of the chaff tip are indicated by red curves in Figure 5. Points L, L_u_, L_d_, K, K_u_, and K_d_ (Figure 6) on the concaves were crucial for feature extraction. The hull points L_d_, L_u_, K_u_, and K_d_ of the seed contour were obtained using the convex hull algorithm [10].

**Figure 7:**
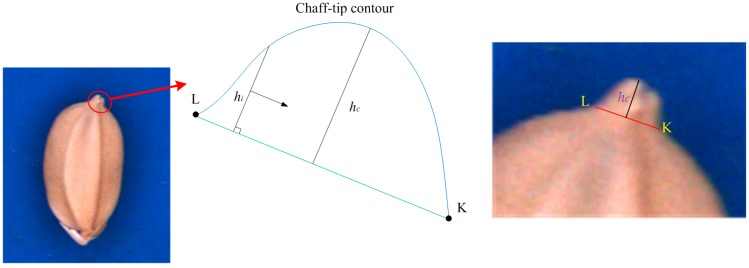
Width and height of chaff tip.

**Figure 8:**
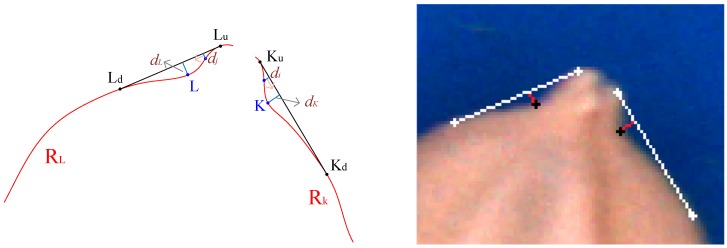
Depths of concaves.

**Figure 9:**
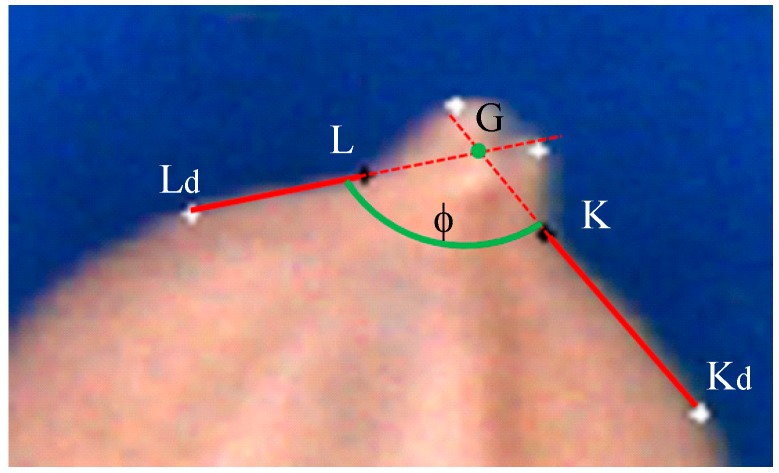
Interior angle φ described by concaves.

Geometric features analysis was employed extensively for classification. In this study, seven features were extracted using the developed algorithm. The feature definition and extraction method proceeded as follows:

(1) $\bar{\mathrm{AB}}$ is the longest line segment on the seed contour.
(2) $\bar{\mathrm{CD}}$ is the perpendicular bisector of $\bar{\mathrm{AB}}$.
(3) The chaff-tip width ($\bar{\mathrm{LK}}$) is as illustrated in Figure 7.
(4) The height (*h_c_* = *max*(*h_i_*)) of the chaff tip is the maximum height of the chaff tip from $\bar{\mathrm{LK}}$, where *d_i_* is the distance between a point on the chaff-tip contour and $\bar{\mathrm{LK}}$, as illustrated in Figure 7.
(5) The depth *d_K_* is the maximum distance between $\bar{K_{u}K_{d}}$ and the concave R_K_ (*d_K_*⊥$\bar{K_{u}K_{d}}$), as indicated in Figure 8. *d_K_* is obtained when *d_K_* = *max*(*d_i_*) at point K.
(6) The depth *d_L_* can be similarly computed, as shown in Figure 8.
(7) The interior angle φ is described by $\bar{{\mathrm{KK}}_{d}}$ and $\bar{{\mathrm{LL}}_{d}}$ and illustrated in Figure 9.

### 2.4. Classifier

**Figure 10:**
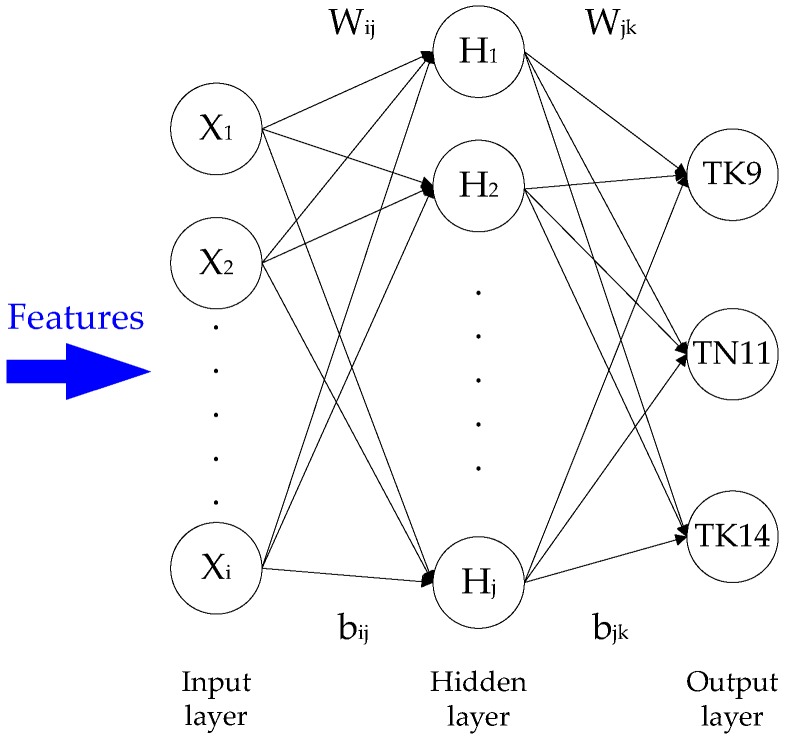
Structure of backpropagation neural network (BPNN) classifier.

In this study, geometric features were employed to differentiate between three paddy seed varieties: TK9, TN11, and TK14. A total of seven geometric features ($\bar{\mathrm{AB}}$, the perpendicular bisector $\bar{\mathrm{CD}}$, the chaff-tip width $\bar{\mathrm{LK}}$ and height, the depths *d_K_*and *d_L_*, and the interior angle φ) were applied in a BPNN [11]. The BPNN classifier consisted of input, hidden, and output layers. The input features were normalized between 0 and 1. The output layer was composed of nodes related to the three categories: TK9, TN11, and TK14. The number of nodes in the hidden layer ($n_{h}$) was calculated using the following formula [12]:

$$n_{h}=n_{i}+n_{o}+k$$

where *n_i_* and *n_o_* are the number of input and output nodes, respectively, *k* = −2, 0, 2. The structure of the BPNN classifier is illustrated in Figure 10, wherein W_ij_ and b_ij_ are the weight and bias of the input layer in the hidden layer and W_jk_ and b_jk_ are the weight and bias of the hidden layer in the output layer. X_i_, H_j_, and O_k_ denote the input layer, hidden layer, and output layer values, respectively.

After its structure was determined, the BPNN classifier was trained. The purpose of BPNN training is to identify relationships between patterns composed of features in each variety of paddy seed. During training, the BPNN classifier analyzed training samples at a given learning rate, and its weights and biases were adjusted until the mean squared error was less than the tolerance error, which indicated that the BPNN classifier had completed training and its weights and biases were stable. In this study, the BPNN classifier analyzed 500 training samples of each variety at a learning rate of 0.01 before training was complete, as defined by a tolerance error of 0.01.

## 3. Results and Discussion

The identifying software for the paddy seeds was developed using Visual Basic 6.0 and MIL 8.0. The functions of software include file operations (acquire, load, and save images), image analysis operations (i.e., binary operator, hole-filling, remove noises using closing, opening, and smoothing), the feature extraction, and BPNN. The variety of paddy can be identified by the software computation accurately and rapidly.

In this study, 1,156 paddy seeds of variety TK9, 1,180 paddy seeds of variety TN11, and 1170 paddy seeds of variety TK14 were used as experimental samples. Of these, 500 seeds of each variety were used as training samples to establish the BPNN classifier, and the remainder was used to test the BPNN classifier. Overfitting often occurs when the training set contains some incorrect samples in the BPNN. However, because the varieties of seed in the training samples were known prior to the training process, overfitting was unlikely to occur here.

The classification accuracies of the BPNN are presented in Table 1 and Table 2, obtained using the TK9, TN11, and TK14 test samples when the number of nodes in the hidden layer was 8, 10, or 12. The accuracy of the BPNN was highest when the number of nodes was 10; nevertheless, the results were also mostly accurate when there were 8, 10, or 12 hidden nodes. We also compared the BPNN with a Bayesian classifier [9] using the same samples and features, the results of which are presented in Table 2. The BPNN’s accuracy was slightly higher than that of the Bayesian classifier for samples TN11 and TK14, but the Bayesian classifier performed favorably for samples TK9. Thus, the BPNN and Bayesian classifiers have the same classification ability for the TK9, TN11, and TK14 varieties. As mentioned previously, the features employed in this study can classify the paddy varieties using different classifiers.

The accuracy of the BPNN when presented with variety TK9 was the lowest, probably because the correlation between the adopted features and the TK9 seed’s contour was not sufficiently strong in this study.

## 4. Conclusions

In this study, we developed a novel method for classifying three varieties (Taikong 9, Tainan 11, and Taikong 14) of foundation paddy seeds. The shape features of the seeds were obtained to establish a BPNN classifier. The test results show that three varieties of foundation paddy seeds can be classified efficiently with this method. In a future study, we intend to further refine the classification algorithm or use other classifiers to increase the seed classification accuracy.

## Figures and Tables

**Table 1:** Results using BPNNs.

***n_h_* = 8**			
**Variety**	**TK9**	**TN11**	**TK14**
TK9	606	4	15
TN11	23	665	15
TK14	27	11	640
Classification Accuracy (%)	92.38	97.79	95.52
Average accuracy (%)	95.26		
***n_h_* = 10**			
**Variety**	**TK9**	**TN11**	**TK14**
TK9	608	5	13
TN11	21	662	10
TK14	27	13	647
Classification Accuracy (%)	92.68	97.35	96.57
Average accuracy (%)	95.56		
***n_h_* = 12**			
**Variety**	**TK9**	**TN11**	**TK14**
TK9	608	5	12
TN11	21	662	15
TK14	27	13	643
Classification Accuracy (%)	92.68	97.35	95.97
Average accuracy (%)	95.36		

**Table 2:** Results using Bayes classifier.

Variety	TK9	TN11	TK14
TK9	613	8	10
TN11	25	651	10
TK14	18	21	646
Total	656	680	670
Classification accuracy (%)	93.44	95.7	96.4
Average accuracy (%)	95.21		
